# Spontaneous Bilateral Internal Carotid Artery Dissections in a Young Female with Headache

**DOI:** 10.5811/cpcem.2016.11.31980

**Published:** 2017-01-24

**Authors:** Jonathan T Jaffe, Thompson Kehrl

**Affiliations:** Wellspan York Hospital, Department of Emergency Medicine, York, Pennsylvania

## Abstract

Spontaneous cervical artery dissection (sCAD) occurs when the intimal lining separates from the outer wall of the artery. Although rare, it is a common cause of stroke in young people. Presentations range from isolated headache to severe stroke symptoms. A 41-year-old woman with minimal past medical history presented with left-sided headache and transient right leg weakness and numbness. The patient underwent computed tomography (CT) angiography of the neck that showed bilateral internal carotid artery dissections with a relative stenosis from pseudoaneurysm formation on the left. She was placed on a heparin drip and transitioned to warfarin but subsequently required stent placement 10 days later. If this patient had not undergone CT angiography at the time of presentation, she might have suffered significant morbidity and possible mortality.

## INTRODUCTION

Spontaneous cervical arterial dissection (sCAD) is a rare but clinically important entity as it causes a significant proportion of strokes in young people. Dissection is found to be the underlying etiology of stroke in 0.4 to 4% of patients but as high as 20% in stroke patients younger than 30 years of age.^1^ The pathophysiology of sCAD involves a tear in the intimal lining of the artery that leads to luminal stenosis or occlusion, intra-arterial thrombus formation, and ultimately stroke via either hypoperfusion or thromboembolism. The carotid or vertebral arteries can be affected, with the former being twice as common as the latter.^2^ The total incidence of spontaneous internal carotid artery dissection (sICAD) is estimated at around three per 100,000,^3^ but the exact incidence is unknown as presentations can range from asymptomatic to isolated headache or neck pain to transient ischemic attack to stroke. The incidence of bilateral carotid dissections is considered rare and is not fully known, but may be as high as 22% of sICADs.^4^,^5^ We present a case of bilateral sICAD presenting with unilateral headache and blurry vision.

## CASE REPORT

A 41-year-old female presented to the emergency department (ED) with four hours of severe sudden-onset left-sided retro-orbital headache. It was associated with blurry vision in her left eye and did not change in intensity. She initially delayed seeking medical evaluation but came to the ED when her symptoms persisted. After the headache had been present for two hours, she noted some altered sensation throughout her right lower extremity but had no complaints of focal weakness. Her medical history was only significant for depression and daily tobacco use. She did not have a history of headaches and there was no trauma.

The patient was evaluated by a physician at triage upon initial arrival in the department and was not noted to have any focal deficits. Her initial vital signs were only notable for a blood pressure of 125/95mmHg. She was evaluated by the treating physicians approximately 20 minutes after arrival and noted to have an NIH stroke scale score of three for slight flattening of the left nasolabial fold, drift in the right leg, and altered sensation in the right leg. Sensation was tested by comparing the perception of sharp stimuli between each lower extremity, which was reported to be more dull throughout the right lower extremity compared to the left in a non-dermatomal pattern. The patient underwent an emergent non-contrast computed tomography (CT) of the head followed by CT angiograms of the neck and brain. The on-call neurologist was consulted and noted no focal deficits on exam with an NIH stroke scale of zero approximately 40 minutes after the exam by the emergency physician. Her imaging studies were reviewed and she was found to have bilateral internal carotid artery dissections with pseudoaneurysm formation on the left creating a relative stenosis (Image).

After discussion with the neurosurgical service, she was started on a heparin drip and admitted to the ICU for monitoring. She underwent cerebral angiography but did not require any intervention at the time, as good flow was seen distal to the dissections and pseudoaneurysm. She was discharged home three days later on an enoxaparin bridge to warfarin.

She re-presented to the ED 10 days later with right-sided numbness that developed into aphasia and right-sided weakness. Her initial complaint was tingling on the right side of her face and along the right arm but this progressed into severe aphasia, dysarthria, diminished strength throughout the right upper and lower extremities, and decreased response to painful stimuli on the right side while she was in the ED. She was taken back to the angiography suite and a stent was placed in the left internal carotid artery. She was discharged three days later on aspirin and clopidogrel. A follow-up CT angiogram of the neck performed four weeks later showed a stable right internal carotid artery dissection and patent left-sided carotid stent.

## DISCUSSION

sCADs cause a large proportion of ischemic strokes in young people, especially in those without known cardiovascular risk factors, with an average age of 45.^6^ Reported incidence of sCAD is likely underestimated as some patients can present with minimal or even no clinical signs.^5^–^8^ In fact, the most common presenting symptom is unilateral frontal headache.^9^ As such, many patients with headache may not undergo CT angiography to evaluate for dissections. Although the diagnosis is difficult because of the variation in presentation, it is a crucial diagnosis to make in the ED with estimates of long-term neurologic sequelae of 30% and mortality of up to 2%.^2^

In the case presented above, the patient’s focal neurologic signs were transient and were not identified on two of her three examinations, including by the attending neurologist, which highlights how easily this diagnosis can be missed. A study by Lee et. al. found transient ischemic attack (TIA) in 23% of sCADs and stroke in 56%.^6^ Associated risk factors for sCAD include smoking, migraines, and fibromuscular dysplasia (FMD).^6^ Horner syndrome is found in 25% of patients with sCAD^6^ and is slightly more common when pseudoaneurysm is present. Hassan et. al. found pseudoaneurysms in 90% of multiple dissections.^5^

There is little in the medical literature about multiple cervical artery dissections. The incidence ranges from 13–22% of all sCADs, but these studies are limited to small sample sizes.^5^,^6^ Signs and symptoms of bilateral sCADs can range from stroke to TIA to only headache, making diagnosis difficult.^8^,^10^ Either or both sides may be symptomatic. Our patient was only symptomatic of her left-sided dissection at the time of presentation. Diagnosis is confirmed with imaging, which can include ultrasound, magnetic resonance imaging or magnetic resonance angiography, or CT angiography.^11^

Management typically includes anticoagulation or antiplatelet therapy, with endovascular intervention having emerged as an additional or alternative therapeutic option.^8^,^10^,^12^–^14^ The Cervical Artery Dissection in Stroke Study (CADISS), published in 2015, compared the use of antiplatelet therapy and anticoagulation in the long-term treatment of stroke secondary to CAD and found no significant difference between the two. Antiplatelet regimens were not uniform and consisted of aspirin, dipyridamole, or clopidogrel, alone or in combination.^13^ No definitive guidelines exist regarding the role of endovascular therapy, but a systematic review out of China suggested stent therapy for patients with recurrent symptoms despite adequate medical therapy, significant cerebral hypoperfusion, a symptomatic or expanding pseudoaneurysm, or a contraindication to anticoagulation.^14^ Since there is no consensus on medical versus endovascular treatments or even a specific ideal medical regimen, current practices will likely vary and rely on discussions between emergency physicians, specialists, and patients. Our patient was managed with stenting on her return visit after previously being treated with anticoagulation, consistent with the current literature.

## CONCLUSION

In conclusion, sCAD is a rare but potentially deadly phenomenon that needs to be considered in patients with headache and neck pain, particularly in the presence of neurologic symptoms. Bilateral dissections, in particular, carry increased risk by making larger regions of the brain susceptible to ischemia via occlusion and thromboemboli. CT angiography does not need to be performed on all patients with head or neck pain, but should be performed when focal neurological complaints or findings are noted and strongly considered when patients report neurologic symptoms prior to arrival.

## Figures and Tables

**Image:**
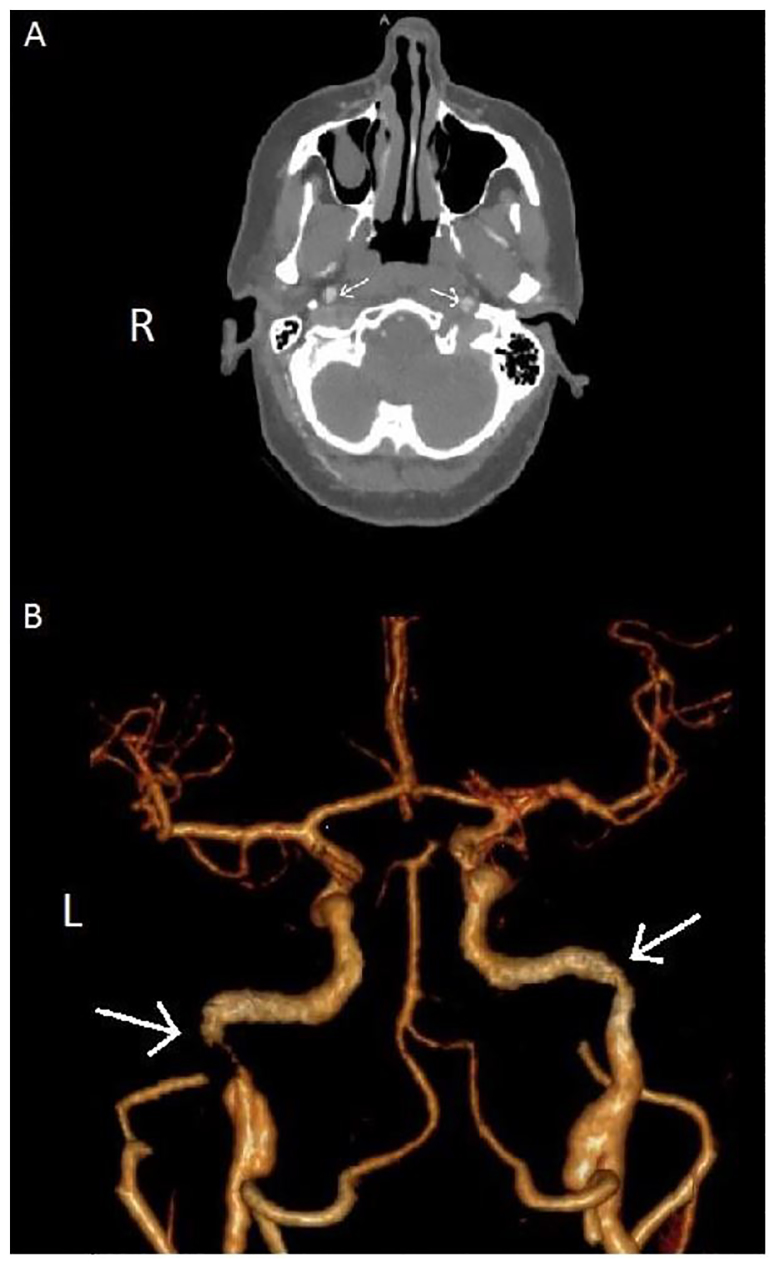
A. Axial CT angiography. Arrows mark intimal flaps in right and left internal carotid arteries. B. 3D reconstruction of CT angiography of the brain with luminal narrowing of bilateral internal carotid arteries marked by arrows. *CT,* computed tomography
